# Supplementary material for: Genomic selection for salinity tolerance in japonica rice
Source: PLoS One. 2023 Sep 27;18(9):e0291833. doi: 10.1371/journal.pone.0291833 (PMC10530037; doi:10.1371/journal.pone.0291833)
Supplement: S1 Fig — Distribution in the rice genome of the informative markers for the complete set of 20,255 SNPs (upper panel) and the non-redundant set of 16,993 SNPs (lower panel). (PDF) [file pone.0291833.s001.pdf]

Supporting information

Genomic selection for salinity tolerance in japonica rice

Bartholomé J[1,2,3*], Frouin J[2,4], Brottier L[2,4], Cao TV[2,4], Boisnard A[5], Ahmadi N[2,4], Courtois B[2,4]

[1] CIRAD, UMR AGAP, Recta Palmira Cali, Colombia
[2] AGAP, Univ Montpellier, CIRAD, INRA, Montpellier SupAgro, Montpellier, France
[3] Alliance Bioversity-CIAT, Recta Palmira Cali, Colombia
[4] CIRAD, UMR AGAP, F-34398 Montpellier, France.
[5] Centre Français du Riz, 13200, Arles

***** Corresponding author
e-mail: jerome.bartholome@cirad.fr
orcid.org/0000-0002-0855-3828

The following Supporting Information is available for this article:

S1 Fig. Distribution in the rice genome of the informative markers for the complete set of 20,255 SNPs (upper panel) and the non-redundant set of 16,993 SNPs (lower panel).

S2 Fig. Distribution of minor allele frequency (MAF) for the 16,993 non-redundant SNPs in the two populations: the reference panel and the breeding population.

S3 Fig. Unweighted neighbor-joining tree and the associated genetic structure for the reference panel and the breeding population. In red the temperate *japonica*, in blue the tropical *japonica* and in purple the admixed accessions between these two groups

S4 Fig. Boxplot for the stress response indices (iTrait) and the K and Na mass fractions and their ratio in the reference panel. The different subpopulations (admixed, temperate, tropical) were defined with molecular markers (see materials and methods). Different letters for a given trait indicate a significant difference between group means (Tukey's HSD test, $p < 0.05$).

S5 Fig. Boxplot of genomic estimated breeding value (GEBV) for the eight morphological traits in the breeding population of 393 lines. The 41 lines selected for the validation experiment are represented in green and the rest of the population is shown in gray. Two prediction methods (GBLUP and RKHS) and two models (single- and multi-environment) were compared.

S6 Fig. Distribution of genomic estimated breeding value (GEBV) for Na and K mass fraction and their ratio (Na/K) in the breeding population of 393 lines. The 41 breeding lines selected for the validation experiment are represented in green and the rest of the population is shown in gray. Two different prediction methods were used: GBLUP and RKHS.

S7 Fig. Assessment of salinity tolerance under hydroponic conditions. The image at the top represents half the 12 tanks for one replicate for the reference panel. The image at the bottom represents the three replicates for the selected genotypes of the breeding population. The control tanks are shown on the left and those for salt conditions are shown on the right.

S1 Table. List of accessions in the two populations: the reference panel and the breeding population. SEE SEPARATE FILE

S2 Table. Correlations between morphological traits in control conditions (upper table) and between ion mass fractions in salt conditions (lower table). The Spearman rank correlation coefficients are displayed in the lower part of the matrices and the associated *p*-value are shown in the upper part.

S3 Table. Analysis of variance of predictive abilities in the reference panel for performances in both sets of conditions (CTRL and SALT). Two prediction methods were compared (GBLUP and RKHS), for eight traits and two models (single- and multi-environment).

S4 Table. Analysis of variance of predictive abilities in the reference panel for indices and ion mass fractions (referred to as Trait in the table). Two prediction methods were compared (GBLUP and RKHS).

S5 Table. Spearman's rank correlation coefficient for the relationship between the predicted performances estimated with RKHS and GBLUP for the entire breeding population, with single- and multi-environment models.

S6 Table Relationship between predictive abilities estimated by cross-validation on the reference panel and those estimated with the subset (41 lines) of the breeding population. Two models (single- and multi-environment) and two methods (GBLUP and RKHS) were evaluated.

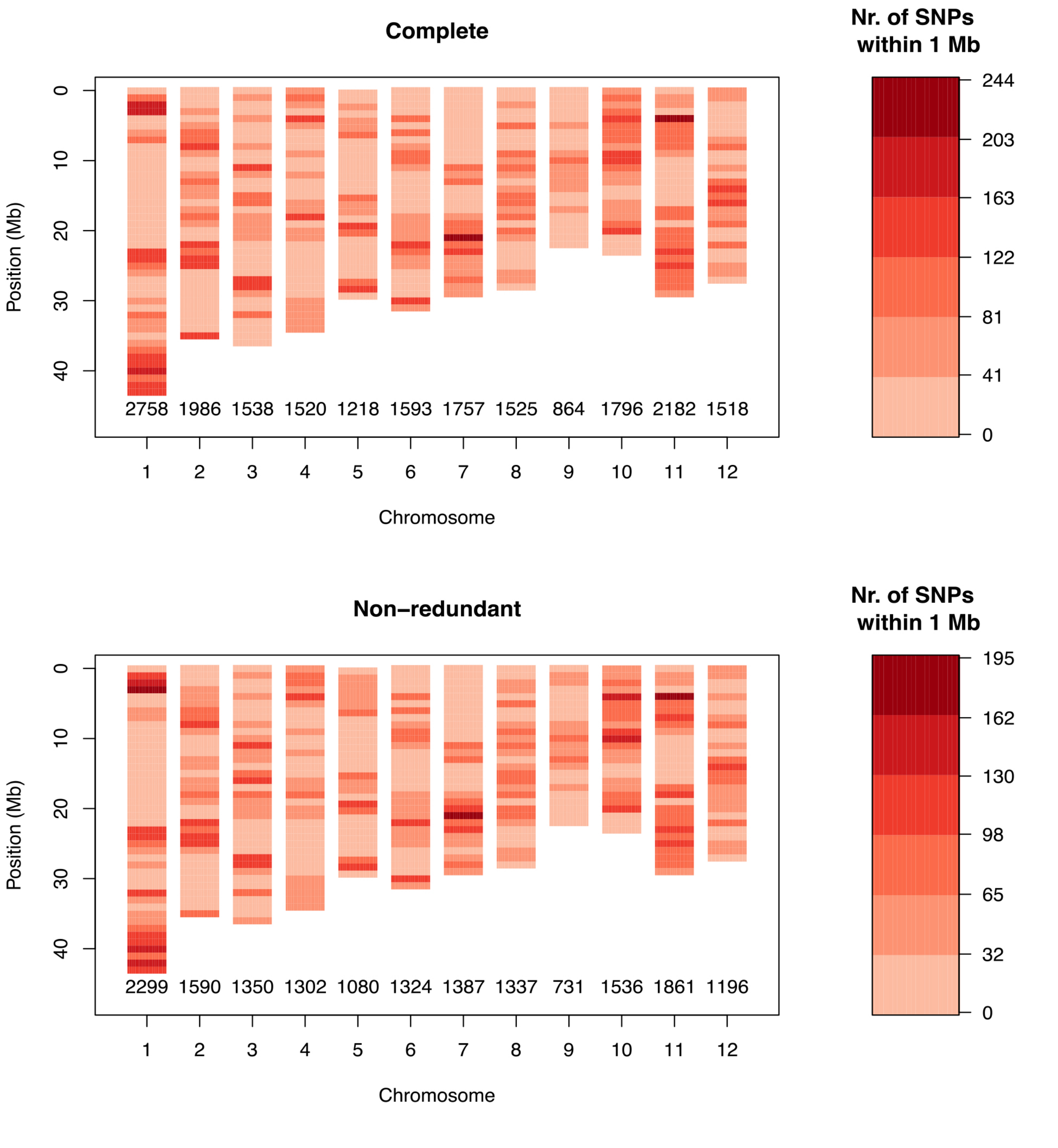

S1 Fig. Distribution in the rice genome of the informative markers for the complete set of 20,255 SNPs (upper panel) and the non-redundant set of 16,993 SNPs (lower panel).
