# Supplementary material for: Genomic selection for salinity tolerance in japonica rice
Source: PLoS One. 2023 Sep 27;18(9):e0291833. doi: 10.1371/journal.pone.0291833 (PMC10530037; doi:10.1371/journal.pone.0291833)
Supplement: S2 Fig — (PDF) [file pone.0291833.s002.pdf]

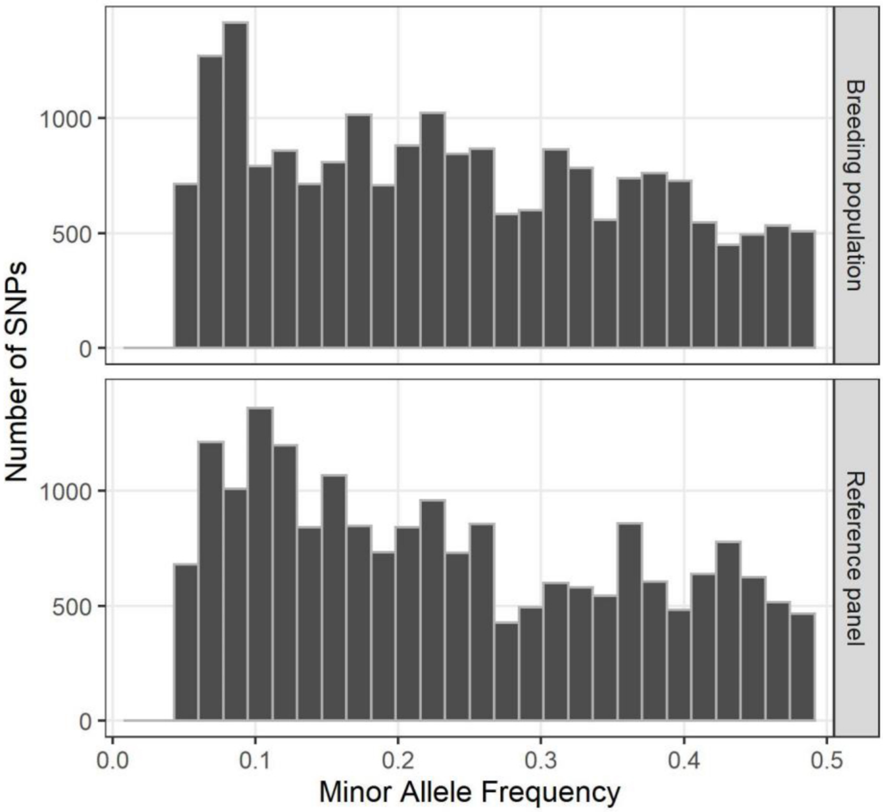

S2 Fig. Distribution of minor allele frequency (MAF) for the 16,993 non-redundant SNPs in the two populations: the reference panel and the breeding population.
