# Supplementary material for: Genomic selection for salinity tolerance in japonica rice
Source: PLoS One. 2023 Sep 27;18(9):e0291833. doi: 10.1371/journal.pone.0291833 (PMC10530037; doi:10.1371/journal.pone.0291833)
Supplement: S3 Fig — Temperate japonica is shown in red, tropical japonica in blue and admixed accessions are shown in purple. (PDF) [file pone.0291833.s003.pdf]

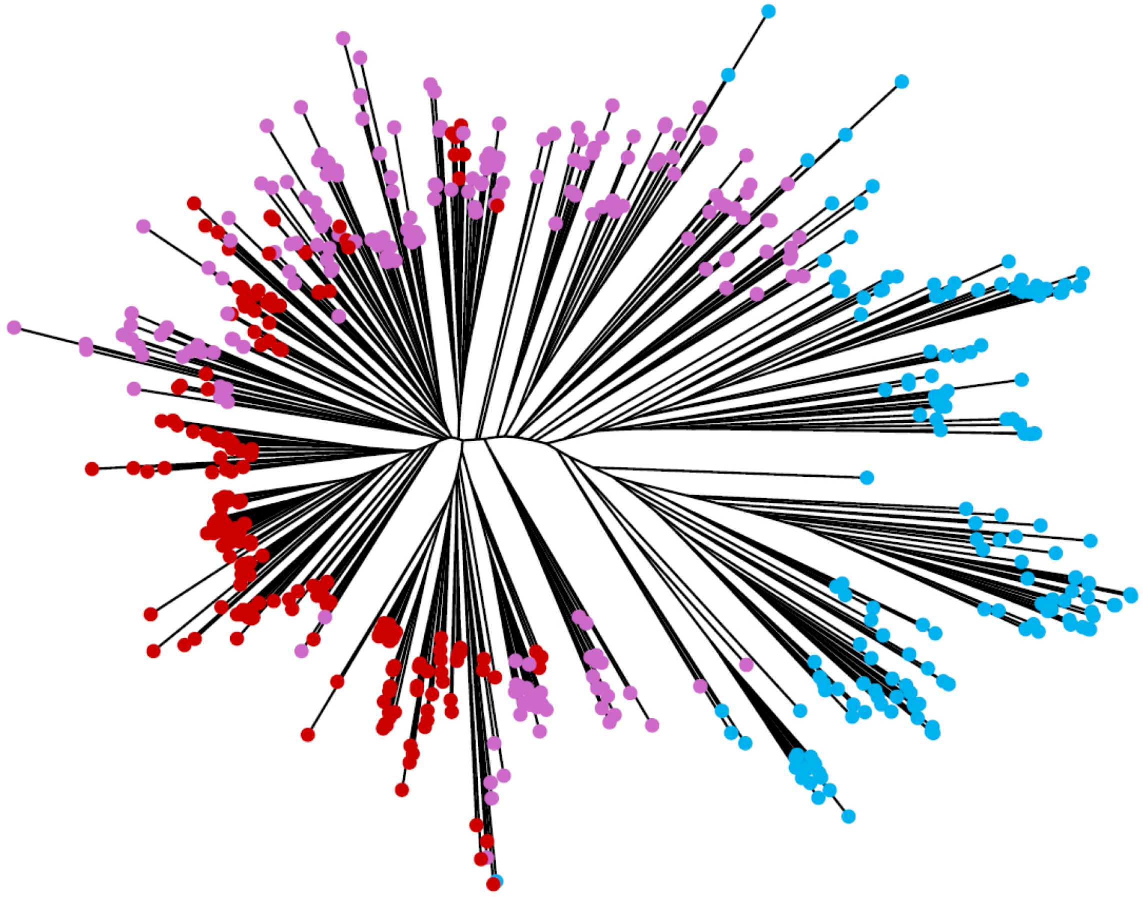

S3 Fig. Unweighted neighbor-joining tree and the associated genetic structure for the reference panel and the breeding population. In red the temperate *japonica*, in blue the tropical *japonica* and in purple the admixed accessions between these two groups.
