# Supplementary material for: Genomic selection for salinity tolerance in japonica rice
Source: PLoS One. 2023 Sep 27;18(9):e0291833. doi: 10.1371/journal.pone.0291833 (PMC10530037; doi:10.1371/journal.pone.0291833)
Supplement: S4 Fig — The different subpopulations (admixed, temperate, tropical) were defined with molecular markers (see materials and methods). Different letters for a given trait indicate a significant difference between group means (Tukey’s HSD test, p < 0.05). (PDF) [file pone.0291833.s004.pdf]

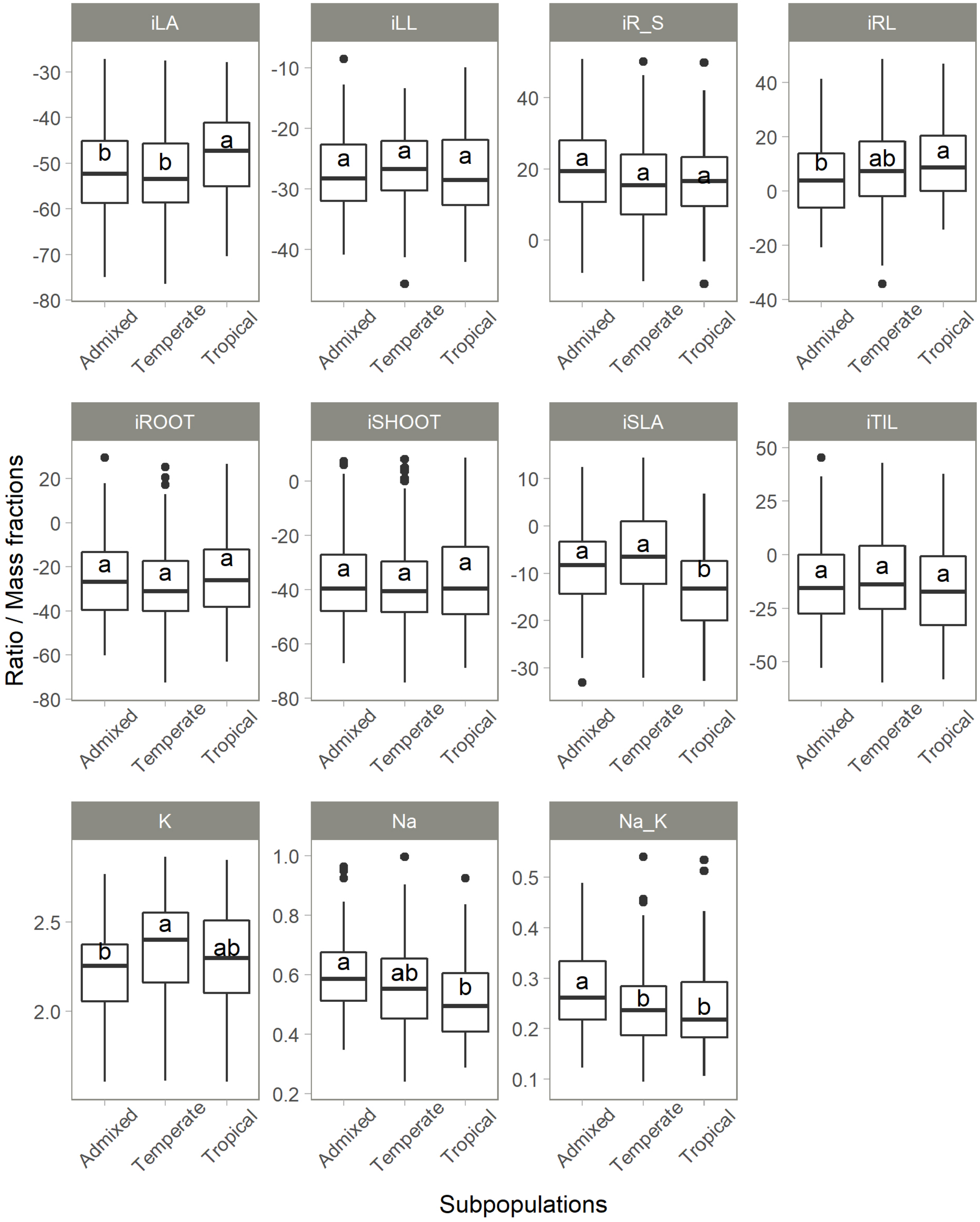

S4 Fig. Boxplot for the stress response indices (iTrait) and the K and Na mass fractions and their ratio in the reference panel. The different subpopulations (admixed, temperate, tropical) were defined with molecular markers (see materials and methods). Different letters for a given trait indicate a significant difference between group means (Tukey's HSD test, $p < 0.05$).
