# Supplementary material for: Genomic selection for salinity tolerance in japonica rice
Source: PLoS One. 2023 Sep 27;18(9):e0291833. doi: 10.1371/journal.pone.0291833 (PMC10530037; doi:10.1371/journal.pone.0291833)
Supplement: S5 Fig — The 41 lines selected for the validation experiment are represented in black and the rest of the population is shown in gray. Two prediction methods (GBLUP and RKHS) and two models (single- and multi-environment) were compared. (PDF) [file pone.0291833.s005.pdf]

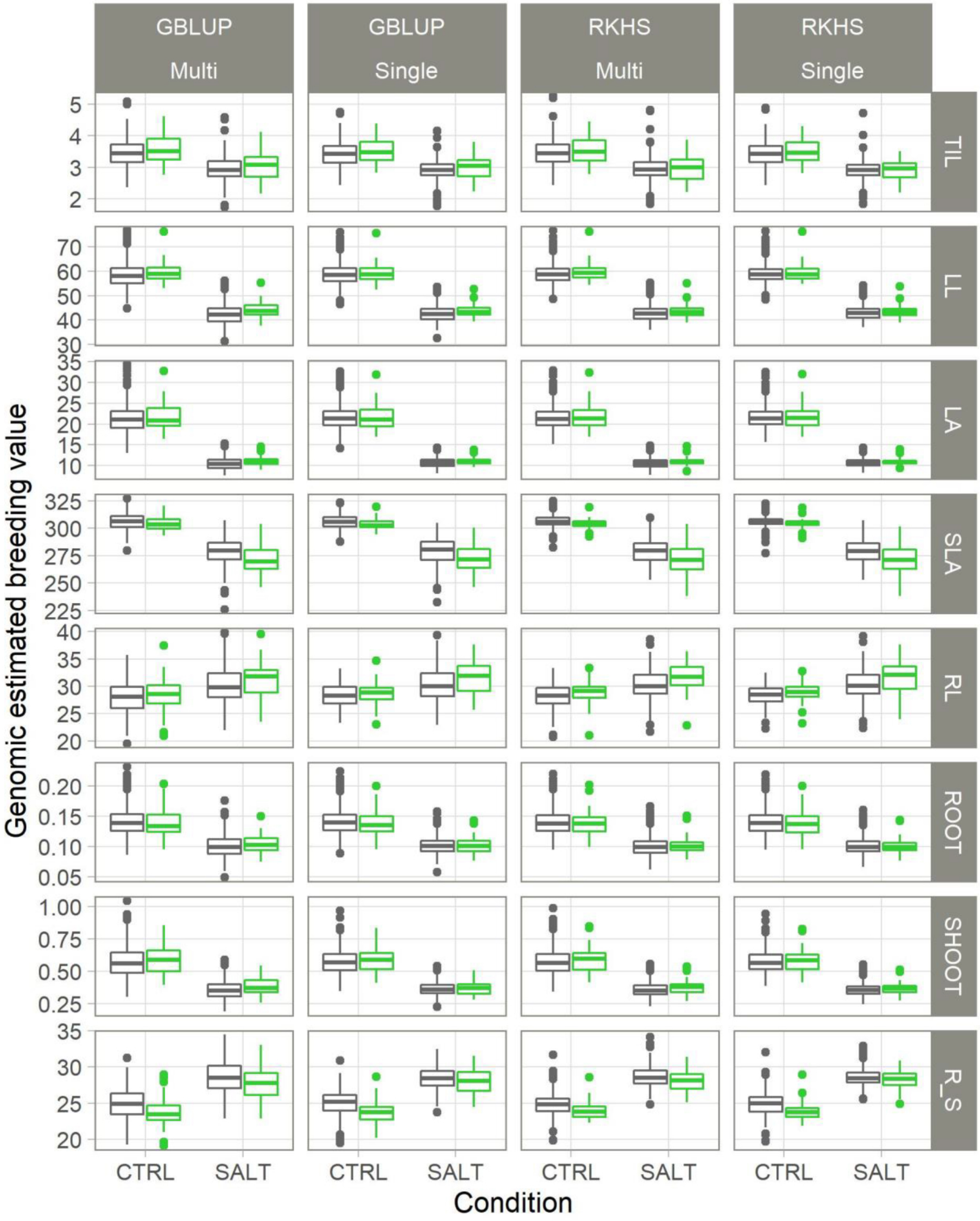

S5 Fig. Boxplot of genomic estimated breeding value (GEBV) for the eight morphological traits in the breeding population of 393 lines. The 41 lines selected for the validation experiment are represented in green and the rest of the population is shown in gray. Two prediction methods (GBLUP and RKHS) and two models (single- and multi-environment) were compared.
