# Supplementary material for: Genomic selection for salinity tolerance in japonica rice
Source: PLoS One. 2023 Sep 27;18(9):e0291833. doi: 10.1371/journal.pone.0291833 (PMC10530037; doi:10.1371/journal.pone.0291833)
Supplement: S6 Fig — The 41 breeding lines selected for the validation experiment are represented in green and the rest of the population is shown in gray. Two different prediction methods were used: GBLUP and RKHS. (PDF) [file pone.0291833.s006.pdf]

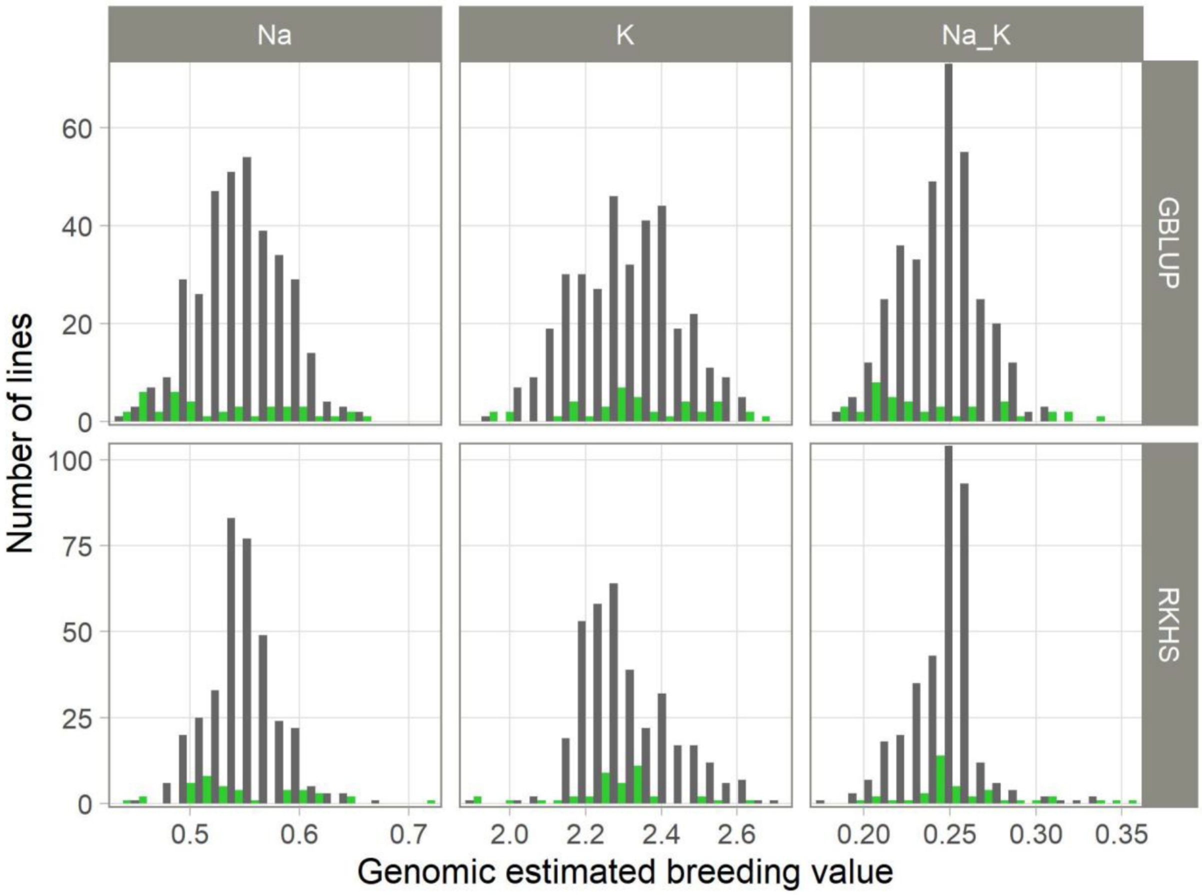

S6 Fig. Distribution of genomic estimated breeding value (GEBV) for Na and K mass fraction and their ratio (Na/K) in the breeding population of 393 lines. The 41 breeding lines selected for the validation experiment are represented in green and the rest of the population is shown in gray. Two different prediction methods were used: GBLUP and RKHS.
