# Supplementary material for: Genomic selection for salinity tolerance in japonica rice
Source: PLoS One. 2023 Sep 27;18(9):e0291833. doi: 10.1371/journal.pone.0291833 (PMC10530037; doi:10.1371/journal.pone.0291833)
Supplement: S7 Fig — The image at the top represents half the 12 tanks for one replicate for the reference panel. The image at the bottom represents the three replicates for the selected genotypes of the breeding population. The control tanks are shown on the left and those for salt conditions are shown on the right. (PDF) [file pone.0291833.s007.pdf]

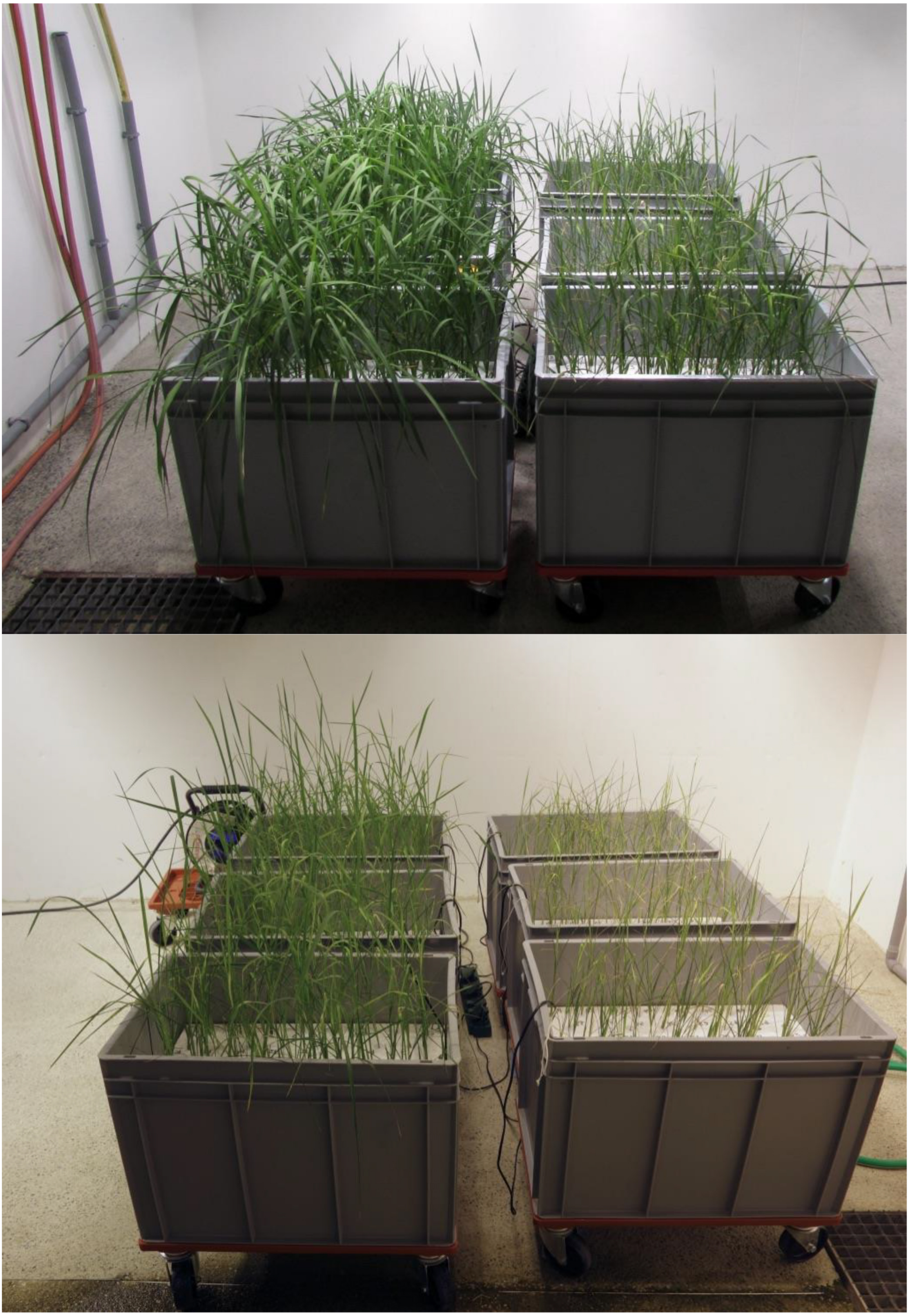

S7 Fig. Assessment of salinity tolerance under hydroponic conditions. The image at the top represents half the 12 tanks for one replicate for the reference panel. The image at the bottom represents the three replicates for the selected genotypes of the breeding population. The control tanks are shown on the left and those for salt conditions are shown on the right.
