# Supplementary material for: Genomic selection for salinity tolerance in japonica rice
Source: PLoS One. 2023 Sep 27;18(9):e0291833. doi: 10.1371/journal.pone.0291833 (PMC10530037; doi:10.1371/journal.pone.0291833)
Supplement: S2 Table — The Spearman rank correlation coefficients are displayed in the lower part of the matrices and the associated p-value are shown in the upper part. (PDF) [file pone.0291833.s009.pdf]

S2 Table. Correlations between morphological traits in control conditions (upper table) and between ion mass fractions in salt conditions (lower table). The Spearman rank correlation coefficients are displayed in the lower part of the matrices and the associated *p*-value are shown in the upper part.

CTRL	TIL	LL	RL	ROOT	SHOOT	LA	SLA	R_S
TIL	1.00	0.00	0.01	0.00	0.00	0.00	0.44	0.00
LL	0.28	1.00	0.00	0.00	0.00	0.00	0.00	0.00
RL	0.17	0.20	1.00	0.00	0.00	0.00	0.13	0.90
ROOT	0.53	0.65	0.28	1.00	0.00	0.00	0.00	0.95
SHOOT	0.69	0.68	0.24	0.90	1.00	0.00	0.00	0.00
LA	0.32	0.77	0.19	0.76	0.76	1.00	0.04	0.00
SLA	0.05	-0.34	-0.10	-0.25	-0.21	-0.14	1.00	0.56
R_S	-0.45	-0.24	0.01	0.00	-0.37	-0.19	-0.04	1.00

SALT	TIL	LL	RL	ROOT	SHOOT	LA	SLA	R_S	Na	K	Na_K
TIL	1.00	0.05	0.01	0.00	0.00	0.02	0.17	0.00	0.03	0.00	0.00
LL	0.13	1.00	0.11	0.00	0.00	0.00	0.00	0.04	0.31	0.21	0.31
RL	0.17	0.10	1.00	0.00	0.01	0.00	0.00	0.02	0.59	0.64	0.87
ROOT	0.55	0.56	0.24	1.00	0.00	0.00	0.00	0.00	0.73	0.00	0.08
SHOOT	0.63	0.64	0.16	0.90	1.00	0.00	0.00	0.02	0.06	0.00	0.00
LA	0.15	0.72	0.21	0.58	0.62	1.00	0.02	0.53	0.22	0.00	0.09
SLA	0.09	-0.37	-0.18	-0.21	-0.22	-0.15	1.00	0.84	0.03	0.03	0.42
R_S	-0.19	-0.13	0.15	0.25	-0.15	-0.04	-0.01	1.00	0.00	0.50	0.00
Na	-0.15	-0.07	-0.04	-0.02	-0.12	-0.08	0.14	0.28	1.00	0.00	0.00
K	0.26	0.08	0.03	0.24	0.25	0.18	0.14	-0.04	-0.31	1.00	0.00
Na_K	-0.20	-0.07	-0.01	-0.12	-0.19	-0.11	0.05	0.22	0.92	-0.60	1.00
