# Supplementary material for: Genomic selection for salinity tolerance in japonica rice
Source: PLoS One. 2023 Sep 27;18(9):e0291833. doi: 10.1371/journal.pone.0291833 (PMC10530037; doi:10.1371/journal.pone.0291833)
Supplement: S3 Table — Two prediction methods were compared (GBLUP and RKHS), for eight traits and two models (single- and multi-environment). (PDF) [file pone.0291833.s010.pdf]

S3 Table. Analysis of variance of predictive abilities in the reference panel for performances in both sets of conditions (CTRL and SALT). Two prediction methods were compared (GBLUP and RKHS), for eight traits and two models (single- and multi-environment).

Source	Sum of squares	Degrees of freedom	F-value	Pr(>F)
Method	1.58	1	91.706	<0.0001
Trait	388.6	7	3230.1368	<0.0001
Model	1.25	1	72.5272	<0.0001
Conditions	3.77	1	219.429	<0.0001
Method:Trait	1.39	7	11.5438	<0.0001
Method:Model	0.22	1	12.8393	0.0003
Trait:Model	1.02	7	8.4435	<0.0001
Method:Conditions	0.83	1	48.0203	<0.0001
Trait:Conditions	183.22	7	1522.9642	<0.0001
Model:Conditions	0.01	1	0.5005	0.4793
Method:Trait:Model	0.25	7	2.0514	0.0452
Method:Trait:Conditions	1.15	7	9.5256	<0.0001
Method:Model:Conditions	0	1	0.2445	0.62109
Trait:Model:Conditions	0.66	7	5.4691	<0.0001
Residuals	548.98	31943		
Total	1132.93	31999		
