# Supplementary material for: Genomic selection for salinity tolerance in japonica rice
Source: PLoS One. 2023 Sep 27;18(9):e0291833. doi: 10.1371/journal.pone.0291833 (PMC10530037; doi:10.1371/journal.pone.0291833)
Supplement: S4 Table — Two prediction methods were compared (GBLUP and RKHS). (PDF) [file pone.0291833.s011.pdf]

S4 Table. Analysis of variance of predictive abilities in the reference panel for indices and ion mass fractions (referred to as Trait in the table). Two prediction methods were compared (GBLUP and RKHS).

Source	Sum of squares	Degrees of freedom	F-value	Pr(>F)
Method	0.309	1	20.118	<0.0001
Trait	146.444	10	953.245	<0.0001
Method:Trait	14.448	10	94.049	<0.0001
Residuals	168.651	10978		
Total	329.852	10999		
