# Supplementary material for: Genomic selection for salinity tolerance in japonica rice
Source: PLoS One. 2023 Sep 27;18(9):e0291833. doi: 10.1371/journal.pone.0291833 (PMC10530037; doi:10.1371/journal.pone.0291833)
Supplement: S5 Table — (PDF) [file pone.0291833.s012.pdf]

S5 Table. Spearman's rank correlation coefficient for the relationship between the predicted performances estimated with RKHS and GBLUP for the entire breeding population, with single- and multi-environment models.

Model	Condition	Trait	Spearman's rho	p-value
Multi	CTRL	TIL	0.98	$<10^{-5}$
		LL	0.93	$<10^{-5}$
		LA	0.97	$<10^{-5}$
		SLA	0.88	$<10^{-5}$
		RL	0.89	$<10^{-5}$
		ROOT	0.97	$<10^{-5}$
		SHOOT	0.97	$<10^{-5}$
		R_S	0.93	$<10^{-5}$
	SALT	TIL	0.98	$<10^{-5}$
		LL	0.92	$<10^{-5}$
		LA	0.98	$<10^{-5}$
		SLA	0.95	$<10^{-5}$
		RL	0.91	$<10^{-5}$
		ROOT	0.96	$<10^{-5}$
		SHOOT	0.96	$<10^{-5}$
		R_S	0.91	$<10^{-5}$
Single	CTRL	TIL	0.99	$<10^{-5}$
		LL	0.95	$<10^{-5}$
		LA	0.98	$<10^{-5}$
		SLA	0.76	$<10^{-5}$
		RL	0.95	$<10^{-5}$
		ROOT	0.99	$<10^{-5}$
		SHOOT	0.99	$<10^{-5}$
		R_S	0.96	$<10^{-5}$
	SALT	TIL	0.94	$<10^{-5}$
		LL	0.94	$<10^{-5}$
		LA	0.99	$<10^{-5}$
		SLA	0.96	$<10^{-5}$
		RL	0.95	$<10^{-5}$
		ROOT	0.97	$<10^{-5}$
		SHOOT	0.96	$<10^{-5}$
		R_S	0.88	$<10^{-5}$
