# Supplementary material for: Genomic selection for salinity tolerance in japonica rice
Source: PLoS One. 2023 Sep 27;18(9):e0291833. doi: 10.1371/journal.pone.0291833 (PMC10530037; doi:10.1371/journal.pone.0291833)
Supplement: S6 Table — Two models (single- and multi-environment) and two methods (GBLUP and RKHS) were evaluated. (PDF) [file pone.0291833.s013.pdf]

S6 Table Relationship between predictive abilities estimated by cross-validation on the reference panel and those estimated with the subset (41 lines) of the breeding population. Two models (single- and multi-environment) and two methods (GBLUP and RKHS) were evaluated.

**Model**	**Method**	**Condition**	**Spearman's rho**	**p-value**
Multi	GBLUP	CTRL	-0.126	0.766
		SALT	0.545	0.163
	RKHS	CTRL	-0.09	0.832
		SALT	0.698	0.0541
Single	GBLUP	CTRL	-0.163	0.699
		SALT	0.643	0.0855
	RKHS	CTRL	0	1
		SALT	0.692	0.0573
